# Community pharmacists' practices and perspectives on deprescribing high‐risk psychotropic medicines: National survey findings

**DOI:** 10.1002/bcp.70466

**Published:** 2026-02-04

**Authors:** Monica Jung, Louisa Picco, Aili V. Langford, Rose Laing, Jana Dostal, Suzanne Nielsen

**Affiliations:** ^1^ Monash Addiction Research Centre, Eastern Health Clinical School Monash University Melbourne Victoria Australia; ^2^ Sydney Pharmacy School, Faculty of Medicine and Health The University of Sydney Sydney New South Wales Australia; ^3^ Centre for Medicine Use and Safety, Faculty of Pharmacy and Pharmaceutical Sciences Monash University Parkville Victoria Australia

**Keywords:** benzodiazepines, community pharmacy practice, deprescribing, gabapentinoids, medicinal cannabis, opioids

## Abstract

**Aim:**

To explore the practices, confidence and perspectives of community pharmacists in deprescribing high‐risk psychotropic medicines, including opioid analgesics, benzodiazepine, gabapentinoids and medicinal cannabis.

**Methods:**

An anonymous, cross‐sectional national online survey was conducted between January and April 2025 among Australian community pharmacists. The survey captured data on pharmacist demographics, their workplace (pharmacy) characteristics, their provision of high‐risk psychotropic medicines, and pharmacists' perspectives, confidence and practices related to implementing strategies to support deprescribing of these medicines. Descriptive statistics and logistic regression analyses were conducted to explore the factors associated with initiating discussions with patients about deprescribing.

**Results:**

The sample comprised of 730 pharmacists, representing approximately 12% of all Australian community pharmacies. Approximately three‐quarters indicated their pharmacy received prescriptions every day for opioids (80.6%), benzodiazepines (75.2%) and gabapentinoids (72.1%), whilst fewer than one‐tenth of pharmacies (8.9%) received medicinal cannabis prescriptions every day. Pharmacists working outside of capital cities (i.e. other urban, rural or remote areas; adjusted odds ratio (aOR): 1.33, 95% confidence intervals (CIs): 0.97–1.83), pharmacy managers/owners (aOR: 1.29, 95% CIs: 1.22–1.50) and those with ≥ 15 years of professional experience (OR: 1.57, 95% CIs: 1.17–2.11) had higher odds of initiating discussions on deprescribing psychotropic medicines compared with those working in capital cities, employee pharmacists and those with <15 years of professional experience, respectively.

**Conclusion:**

These findings provide some of the first insights into deprescribing practices of high‐risk psychotropic medicines within community pharmacy settings, highlighting clear opportunities to strengthen these practices, particularly through supporting early‐career pharmacists and those practicing in capital cities.

1 **What is already known about this subject**

- Harms due to high‐risk psychotropic medicines such as opioids and benzodiazepines remain a significant public health concern globally, and more recently, there have been rising concerns about harms due to gabapentinoids and medicinal cannabis prescriptions.
- Pharmacist‐led deprescribing interventions have emerged as a key strategy to mitigate these harms; however, the practices and perspectives of community pharmacists in deprescribing these medicines are relatively unexplored.

**What this study adds**

- This study offers one of the first insights into the role of pharmacists in providing high‐risk psychotropic medicines in community pharmacy settings.
- The use of a national sample of Australian community pharmacists provides a robust picture of their practice, knowledge, perspectives and confidence around supporting deprescribing.
- Findings support the need for targeted support and training, especially for pharmacists based in capital cities, employee pharmacists (as opposed to pharmacy owners/managers) and those with <15 years of professional experience, to support deprescribing.

## 1 INTRODUCTION

Medication‐related harm represents a significant burden to public health globally, contributing to significant rates of injury, ambulance call‐outs, emergency department presentations, hospitalizations, injection‐related harms, overdose and death.^1^, ^2^ Specifically, opioids are the third highest contributor (after tobacco and alcohol) to morbidity and mortality, with more than 70% of the 600 000 drug‐related deaths attributed to opioid misuse globally in 2019.^3^, ^4^ In recent years, high rates of use and related harms from other psychotropic medicines including benzodiazepines, gabapentinoids and medicinal cannabis, have also garnered international concern. Evidence indicates a steady rise in the use of benzodiazepines and gabapentinoids, along with notable non‐medical use and associated harms, particularly in high‐income countries such as the US, Canada and Australia over the past few decades.^5^, ^6^, ^7^ The prescribing of medicinal cannabis, primarily for pain, sleep and mental health conditions, is also prevalent and increasing worldwide.^8^, ^9^ In Australia, prescribing of medicinal cannabis has increased dramatically since the 2016 legalization of cannabis products for medical reasons under the Special Access Scheme and the Authorized Prescriber Scheme of the Therapeutic Goods Administration.^10^ Recent concerns with rising medical cannabis use and related harms including psychosis and development of cannabis use disorder has prompted regulators to review current regulatory arrangements for these products^11^.

Deprescribing, defined as the planned and supervised dose reduction or discontinuation of inappropriate or unnecessary medicines, has emerged as a key strategy to mitigate medication‐related harms.^12^ This is particularly relevant to psychotropic medicines, where evidence supports short‐term benefit but not long‐term use (e.g. long‐term opioid use for chronic non‐cancer pain or long‐term benzodiazepine use for insomnia), making them key therapeutic targets for deprescribing. In clinical practice, deprescribing is conducted collaboratively between the patient and clinicians within the context of the patient's care goals, function, values and preferences.^13^ The process (e.g. determining when and how to deprescribe) varies according to individual patient needs and medication class, with no gold standard approach.^14^, ^15^ It can be complex and challenging, particularly for psychotropic medicines, where physiological dependence and withdrawal symptoms may develop upon discontinuation.^14^, ^16^

Deprescribing is a key initiative that addresses national and international priority areas, including the World Health Organization's Third Global Patient Safety Challenge: ‘Medication Without Harm’^2^ and Australia's National Medicines Policy and the National Strategy for Quality Use of Medicines.^17^, ^18^ Several studies, including a 2025 systematic review examining the role of U.S. pharmacists in deprescribing, demonstrated that pharmacists are well positioned to support deprescribing interventions, given their unique expertise in medication management.^19^, ^20^ In addition, the Canadian professional practice model for pharmacist‐led deprescribing has demonstrated reductions in the use of potentially inappropriate medications, contributing to reduced patient harm and lower healthcare costs.^21^ Furthermore, in response to the aforementioned priorities, several frameworks and guidelines have been developed to support deprescribing. For example, in Australia, an Evidence‐Based Clinical Practice Guideline for Deprescribing Opioid Analgesics, approved by the National Health and Medical Research Council, was published in 2022.^22^ Despite this growing evidence base, several implementation barriers, including time constraints and inadequate education or training, continue to limit the translation of deprescribing frameworks into routine practice.^19^, ^23^

As in many other countries, Australian community pharmacists are positioned at the interface between prescribers and patients and are highly accessible in primary care.^24^ However, despite their accessibility and frequent contact with patients who are prescribed high‐risk psychotropic medicines, there is limited real‐world evidence on the role of community pharmacists in delivering medication safety interventions and supporting deprescribing. In particular, there is a lack of data on Australian community pharmacists' current deprescribing practices, including their confidence and perceived barriers to initiating discussions and implementing deprescribing strategies during routine care.

## 2 AIM

This study aimed to explore the practices, confidence and perspectives of community pharmacists in deprescribing high‐risk psychotropic medicines, including opioid analgesics, benzodiazepines, gabapentinoids and medicinal cannabis.

## 3 METHODS

### 3.1 Study design, participants and setting

Australia has approximately 6000 community pharmacies nationwide across eight states and territories: New South Wales (NSW), Victoria (VIC), Queensland (QLD), Western Australia (WA), South Australia (SA), Tasmania (TAS), Northern Territory (NT) and the Australian Capital Territory (ACT).^25^ Pharmacists working in these settings play a vital role in primary care, through delivering government‐subsidized medicines and health consultations, including screening, monitoring and patient education. To understand pharmacists' practice, attitudes and knowledge on a broad range of harm reduction services, including best practices around deprescribing of high‐risk psychotropic medicines, we conducted a national, cross‐sectional, anonymous online survey of Australian community pharmacists. The survey was conducted via Qualtrics, an online survey tool, between January and April 2025. This study was approved by the Monash University Human Research Ethics Committee (ID: 45196).

### 3.2 Sampling and recruitment

Using publicly available pharmacy marketing lists, we aggregated and randomized the list of all community pharmacies for each state and territory using the ‘=rand()’ formula on Microsoft Excel. We then contacted each pharmacy via telephone, starting from the top of each jurisdiction‐level list, with the aim of including at least 10% from each state and territory, based on the power calculation used in previous literature.^26^ Pharmacists who agreed to participate were provided a link to the online survey via their preferred email address. To avoid intra‐site correlation, we invited only one pharmacist for each pharmacy to participate, and we asked them not to share the link with others. The link included an online participant information sheet and a consent form, which they were required to complete before beginning the survey. Pharmacists were given the opportunity to enter a prize draw to win one of two iPads or one of four $100 e‐gift cards upon completion of the survey as an incentive. Identifiable information required for the prize draw, such as name and email address, were collected separately from the survey responses to ensure anonymity.

### 3.3 Survey measures

The survey content was informed by the current literature on deprescribing high‐risk psychotropic medicines,^15^, ^22^ previous Australian surveys involving community pharmacists,^26^, ^27^ and a recent eDelphi study, which outlined pharmacy‐based best practice for supporting safe use of prescription opioids.^28^ In addition, an expert advisory board was established at the commencement of this study. The advisory board consisted of twelve local and international experts, including researchers, clinicians, consumers and stakeholders from relevant government and professional organizations. Members of the advisory board provided iterative feedback during the survey development process to help ensure the data collected are relevant and applicable to practice, and piloted the final survey to assess the feasibility.

#### 3.3.1 Pharmacy and pharmacist‐in‐charge characteristics

Pharmacists were asked to provide demographic information, including their role (i.e. owner/manager or employee pharmacist), years of professional experience and gender. Pharmacy‐level data collected included state or territory, pharmacy location (i.e. capital city or other urban, rural or remote areas), pharmacy type (i.e. chain/banner group or single independent), average daily script count, staffing levels and availability of private consultation rooms.

#### 3.3.2 High‐risk psychotropic medicines deprescribing practices

Pharmacists were asked a series of questions about four high‐risk psychotropic medication classes: 1) opioid analgesics, 2) benzodiazepines (including benzodiazepine‐like medicines such as zopiclone and zolpidem), 3) gabapentinoids and 4) medicinal cannabis. ‘Deprescribing practice’ was operationalized as self‐reported frequency of deprescribing activities, including dispensing, initiation of discussions on deprescribing and provision of tapering strategies or support. Specifically, pharmacists were asked to indicate, in the past three months, how often they received prescriptions for each of these medication classes: 1) everyday, 2) a few times a week, 3) less often than several times a week and 4) my pharmacy does not supply the medicine. For those who had received prescriptions in the past three months, additional questions on deprescribing practices were asked. Specifically, pharmacists were asked to indicate whether they had initiated discussions on deprescribing (yes/no) and what tapering strategies or support they provided to their patients, if any, for each medication class. These included: 1) liaise with general practitioners (GP) or medical specialists (e.g. pain specialist, addiction medicine specialists), 2) provide counselling and emotional support, 3) help patients connect with allied health professionals (e.g. physiotherapists), 4) provide tapering plans (e.g. a guide for what dose to take each day) and 5) recommend symptomatic medications to help manage withdrawal symptoms.

#### 3.3.3 Pharmacists' perspectives and confidence around deprescribing practices

For pharmacists who indicated that they supplied the medication at their pharmacy, questions were asked to determine their comfort, confidence and beliefs regarding implementation of strategies to support deprescribing in their practice. These included: i) ‘what percentage of patients were you concerned about supplying the following medicines to due to concerns about problematic use or possible dependence or addiction?’, ii) ‘how comfortable do you feel intervening when you are concerned about a prescription for the following medicines (i.e. when you are concerned that use may not be therapeutic or that dependence may be developing)?’ (comfortable/uncomfortable), iii) ‘how confident are you in your ability to provide information on the safe and effective use of the following medicines?’ (confident/not confident), iv) ‘do you think it is part of your role as a pharmacist to discuss: a) risk of opioid overdose, b) risk of benzodiazepine overdose or c) risk of gabapentinoid overdose, with your patients?’ (yes/no/unsure) and v) ‘how confident do you feel about discussing tapering or deprescribing (i.e. supervised process of dose reduction or stopping of controlled medicines) of the following medicines with your patients?’ (confident/not confident).

To ensure study rigour, we included these specific measures, which have been used in various pharmacy studies,^29^, ^30^ and conducted cognitive testing to ensure that pharmacists accurately understood the survey questions and their intended meaning. Comfort and confidence were measured using a four‐point scale (‘very uncomfortable’, ‘uncomfortable’, ‘comfortable’ and ‘very comfortable’; ‘not confident at all’, ‘not confident’, ‘confident’ and ‘very confident’). For the analyses, responses were dichotomised into a binary variable: *comfortable* (‘comfortable’ or ‘very comfortable’) *vs. not comfortable* (‘very uncomfortable’ or ‘uncomfortable’); *confident* (‘confident’ or ‘very confident’) *vs. not confident* (‘not confident at all’ or ‘not confident’).

### 3.4 Data analysis

Descriptive statistics were used to summarize pharmacy and pharmacist‐in‐charge characteristics, as well as pharmacists' practices, confidence and perspectives of deprescribing high‐risk psychotropic medicines. Ordinal logistic regression was used to estimate the adjusted odds ratios (aORs) and 95% confidence intervals (CIs) to examine pharmacy and pharmacist‐in‐charge characteristics associated with initiating discussions on deprescribing of 0 to 4 high‐risk psychotropic medication classes (opioid analgesics, benzodiazepines, gabapentinoids and medicinal cannabis). Additional multivariate logistic regression analysis was conducted to examine whether any opioid‐specific interventions are associated with initiating discussions on deprescribing opioid analgesics. The model included: i) pharmacists' familiarity with opioid health literacy resources (i.e. heard of the Opioid Safety Toolkit [https://saferopioiduse.com.au ^31^; yes/no], ii) use of real‐time prescription drug monitoring program (all the time/less frequently), iii) supply of take‐home naloxone (yes/no) and iv) provision of opioid agonist treatment (yes/no). Data cleaning and analyses were conducted using StataCorp STATA, version 18 BE.^32^

## 4 RESULTS

The study included 730 community pharmacists across Australia, with approximately one‐third from New South Wales (31.4%) and approximately one‐quarter from Victoria (23.8%) and Queensland (20.4%), the country's most populous states. Approximately one‐third were aged between 25–34 years (35.5%) and 35–44 years (30.7%), and most were female (53.0%). Nearly half (44.5%) had ≥15 years of experience in pharmacy practice, and just over half were owners/managers (51.6%) as opposed to being an employee pharmacist (48.4%). Almost one‐third (30.4%) of the pharmacists reported receiving further education or training in deprescribing psychotropic medicines after graduating as a pharmacist, whilst most (84.0%) believed they would benefit from further training in this area.

### 4.1 Supply of high‐risk psychotropic medicines

Almost all of the participants supplied opioid analgesics (99.7%), benzodiazepines (99.9%) and gabapentinoids (99.9%), whilst only 81.6% reported supplying medicinal cannabis at their pharmacy in the past three months (Table 1). Approximately three‐quarters of the pharmacies received prescriptions every day for opioids (80.6%), benzodiazepines (75.2%) and gabapentinoids (72.1%), whilst only 8.9% of pharmacies received medicinal cannabis prescriptions every day.

**TABLE 1:** Pharmacists' perspectives and deprescribing practices for opioid analgesics, benzodiazepines, gabapentinoids, medicinal cannabis.

	Opioid analgesics	Benzodiazepines	Gabapentinoids	Medicinal cannabis
	n (%)	n (%)	n (%)	n (%)
	(n = 730)	(n = 730)	(n = 730)	(n = 730)
Frequency of prescriptions received (past 3 months)				
Everyday	588 (80.6)	549 (75.2)	526 (72.1)	65 (8.9)
A few times a week	123 (16.9)	144 (19.7)	178 (24.4)	161 (22.1)
Less often than several times a week	17 (2.3)	36 (4.9)	25 (3.4)	370 (50.7)
My pharmacy does not supply the medicine	2 (0.3)	1 (0.1)	1 (0.1)	134 (18.4)
Initiated discussions on deprescribing in the past 3 months				
Yes	352 (48.2)	310 (42.5)	222 (30.4)	40 (5.5)
No	378 (51.8)	420 (57.5)	508 (69.6)	690 (94.5)

Of those that supply the medicines in their pharmacies	Opioid analgesics	Benzodiazepines	Gabapentinoids	Medicinal cannabis
	(n = 728)	(n = 729)	(n = 729)	(n = 596)
% of patients pharmacists were concerned about supplying medicines to, due to concerns about problematic use or possible dependence/addiction in the last 3 months (Mean [SD])	19.5% (SD: 19.3)	22.8% (22.6)	17.8% (SD: 20.1)	14.4% (SD: 23.9)
Comfortable intervening when concerned about prescription of medicines *(*i.e. *use may not be therapeutic or that dependence may be developing)*				
Uncomfortable	176 (24.2)	193 (26.5)	171 (23.5)	243 (40.8)
Comfortable	552 (75.8)	536 (73.5)	558 (76.5)	353 (59.2)
Confident in their ability to provide information on the safe and effective use for high‐risk psychotropic medicines				
Not confident	14 (1.9)	19 (2.6)	32 (4.4)	237 (39.8)
Confident	714 (98.1)	710 (97.4)	697 (95.6)	359 (60.2)
Believe it is the pharmacist's role to discuss the risk of overdose				
No/unsure	19 (2.6)	33 (4.5)	51 (7.0)	n/a
Yes	709 (97.4)	696 (95.5)	678 (93.0)	n/a
Confident in discussing tapering or deprescribing with patients				
Not confident	166 (22.8)	183 (25.1)	211 (28.9)	371 (62.3)
Confident	562 (77.2)	546 (74.9)	518 (71.1)	225 (37.8)
Tapering strategies or support provided for patients who are undergoing taper of these medicines				
Liaise with general practitioners or medical specialists (e.g. pain specialist, addiction medicine specialists)	552 (79.7)	532 (78.0)	497 (75.4)	279 (74.2)
Provide counselling and emotional support	516 (74.5)	505 (74.1)	471 (71.5)	252 (67.0)
Help patients connect with allied health professionals	231 (33.3)	206 (30.2)	202 (30.7)	110 (29.3)
Provide tapering plans (i.e. a guide for what dose to take each day)	283 (40.8)	279 (40.9)	250 (37.9)	100 (26.6)
Recommend symptomatic medications to help manage withdrawal symptoms	273 (39.4)	230 (33.7)	201 (30.5)	93 (24.7)

### 4.2 Provision of tapering or deprescribing strategies

More pharmacists reported initiating discussions on deprescribing in the last three months for opioids (48.2%) than for benzodiazepines (42.5%), gabapentinoids (30.4%) and medicinal cannabis (5.8%) (Table 1). Approximately one‐third of the pharmacists did not initiate discussions on deprescribing for any of the four medicines in the last 3 months (37.7%), whilst approximately one‐fifth initiated discussions for three or four medication classes (18.8%) (Supplementary material: Table S1). Among those who supplied high‐risk psychotropic medicines, all participants reported providing at least one tapering strategy or support for patients undergoing taper of those medicines. Liaising with general practitioners or medical specialists was the most frequently reported strategy adopted by pharmacists for supporting tapering, reported at a similar frequency for all four medication classes (opioids (79.7%), benzodiazepines (78.0%), gabapentinoids (75.4%) and medicinal cannabis (74.2%) (Table 1)). The least frequently reported strategies used for tapering opioids and benzodiazepines were helping patients connect with allied health professionals (33.3% and 30.2%, respectively), whilst recommending symptomatic medications to help manage withdrawal symptoms was the least frequently reported strategy for tapering gabapentinoids (30.5%) and medicinal cannabis (24.7%).

### 4.3 Pharmacists' perspectives and confidence around deprescribing practices

Pharmacists were concerned about supplying opioids to 19.5% of their patients in the past 3 months due to concerns about problematic use, dependence, of addiction (Table 1). For benzodiazepines, this was 22.6%, with fewer reporting concerns regarding gabapentinoids (17.8%) and medicinal cannabis (14.4%). Whilst some pharmacists were not *comfortable* intervening when concerned about a prescription (i.e. due to concerns that use may not be therapeutic or that dependence may be developing) for opioids (24.2%), benzodiazepines (26.5%), gabapentinoids (23.5%) and medicinal cannabis (40.8%), most were *confident* in their ability to provide information on the safe and effective use of opioids (98.1%), benzodiazepines (97.4%), gabapentinoids (95.6%) and medicinal cannabis (60.2%).

### 4.4 Correlates of initiating discussions on deprescribing

Adjusting for pharmacy and pharmacist‐level characteristics (including pharmacy location, average daily script count of the pharmacy, number of pharmacists and dispensary technicians/assistants working during weekdays, pharmacist role and years of experience), pharmacists who work in urban, rural or remote areas were more likely to initiate deprescribing discussions across a greater number of medication classes (aOR: 1.33, 95% CIs: 0.97–1.83), compared with those working in capital cities (Table 2). Pharmacists working in pharmacies with an average daily script count between 101 and 200 prescriptions were more likely to initiate discussions compared to those with a script count of less than 100 prescriptions (aOR: 1.68, 95% CIs: 1.09–2.58). However, this association was not significant for those with more than 200 prescriptions. Pharmacists who were managers/owners (aOR: 1.29, 95% CIs: 1.22–1.50) or had ≥15 years of experience in practice (aOR: 1.57, 95% CIs: 1.17–2.11) were more likely to initiate discussions, compared to employee pharmacists or those with <15 years in practice, respectively.

**TABLE 2:** Ordinal logistic regression showing pharmacy and pharmacist‐in‐charge characteristics associated with initiating discussions on deprescribing of 0 to 4 high‐risk psychotropic medication classes (opioid analgesics, benzodiazepines, gabapentinoids and medicinal cannabis).

	Adjusted odds ratio^a^ (95% CI)
**PHARMACY CHARACTERISTICS**	
State/territory	
New South Wales	*Reference*
Victoria	1.07 (0.72,1.60)
Queensland	0.77 (0.51, 1.17)
Western Australia	0.81 (0.47,1.38)
Australian Capital Territory, Northern Territory, Tasmania, South Australia	(0.96, 0.60,1.53)
Pharmacy location	
Capital city	*Reference*
Urban^b^, rural, remote	**1.33 (0.97, 1.83)**
Pharmacy type	
Chain/banner group	*Reference*
Single independent	0.96 (0.70, 1.33)
Average daily script count	
≤ 100	*Reference*
101–200	**1.68 (1.09, 2.58)**
201–300	1.55 (0.90, 2.66)
≥ 300	1.57 (0.63, 1.90)
Number of pharmacists working (weekdays)	
1	*Reference*
2	0.86 (0.60, 1.25)
3+	1.09 (0.63, 1.90)
Number of dispensary technicians and/or assistants	
0	*Reference*
1	1.09 (0.70, 1.67)
2	0.92 (0.56, 1.52)
3+	1.40 (0.78, 2.52)
Private consultation area	
No	*Reference*
Yes	0.95 (0.66, 1.37)
**PHARMACIST‐IN‐CHARGE CHARACTERISTICS**	
Pharmacist role	
Manager/owner	**1.29 (1.22, 1.50)**
Employee pharmacist	*Reference*
Year of practice	
< 15 years	*Reference*
≥ 15 years	**1.57 (1.17, 2.11)**
Gender^c^	
Male	*Reference*
Female	1.12 (0.83, 1.50)

*Note*: Bold values indicate statistical significance at P < 0.05.

^a^ Adjusted for all covariates listed in this table.

^b^ Urban areas excluding capital cities.

^c^ ‘Non‐binary/gender diverse/prefer not to say’ category comprised <0.5% of the sample (not reported in the table).

Additional analysis examining opioid‐specific interventions associated with initiating discussions on opioid deprescribing showed that pharmacists who were familiar with consumer health literacy resources (the ‘Opioid Safety Toolkit’) (aOR: 1.60, 95% CIs: 1.08–2.37) and those using their jurisdiction's prescription drug monitoring program ‘*all the time’* (aOR: 1.46, 95% CIs: 1.02–2.08) were more likely to initiate discussions, compared with those who had not heard of the health literacy resources or used the prescription drug monitoring program less frequently, respectively (Table 3).

**TABLE 3:** Opioid‐specific interventions and overdose prevention strategies associated with initiating discussions on opioid deprescribing.

	*Initiated discussions on opioid deprescribing in the last 3 months*		Adjusted odds ratio^a^ (95% CI)
	Yes	No	
	N = 352 (48.2%)	N = 378 (51.8%)	
**Have you heard of the Opioid Safety Toolkit?**			
No	265 (75.3)	312 (82.5)	*Reference*
Yes	87 (24.7)	66 (17.5)	**1.60 (1.08, 2.37)**
**How often have you used your state's real‐time prescription monitoring program?**			
All the time	225 (63.9)	223 (59.0)	**1.46 (1.02, 2.08)**
Most of the time/sometimes/rarely/never	127 (36.1)	155 (41.0)	*Reference*
**Does your pharmacy stock naloxone?**			
No	94 (26.7)	102 (27.0)	*Reference*
Yes	258 (73.3)	276 (73.0)	0.95 (0.66, 1.36)
**Does your pharmacy offer Opioid Agonist Treatment (OAT) or Pharmacotherapy?** ^**b**^			
No	173 (51.2)	196 (56.3)	*Reference*
Yes	165 (48.8)	152 (43.7)	1.17 (0.85, 1.62)

*Note*: Bold values indicate statistical significance at P < 0.05.

^a^ Adjusted for pharmacy characteristics (including state or territory, pharmacy location, pharmacy size, average daily script count) and pharmacist‐in‐charge characteristics (including pharmacist role, years of practice and gender).

^b^ Missing data (n = 44) due to incomplete survey responses.

## 5 DISCUSSION

Among a national sample of 730 community pharmacists, most reported routinely receiving prescriptions for opioid analgesics, benzodiazepines and gabapentinoids, whilst medicinal cannabis was received far less frequently. Despite this, fewer than half initiated discussions on deprescribing for opioids, benzodiazepines and gabapentinoids in the past three months, and only a small proportion had initiated discussions on deprescribing for medicinal cannabis. These findings may partly reflect the clinical context in which deprescribing is not always appropriate, such as when opioids are prescribed long‐term for cancer pain or palliative care and gabapentinoids for seizure management. Nevertheless, the limited engagement observed may also indicate broader barriers constraining pharmacists' capacity to support deprescribing initiatives.

Interestingly, despite a small proportion of pharmacists initiating discussions on deprescribing, most pharmacists reported being comfortable intervening when concerned about prescribing of opioids, benzodiazepines and gabapentinoids, confident in their ability to provide information on the safe and effective use of those medicines and confident in discussing deprescribing with their patients. Prior research showed that successful delivery of pharmacist‐led deprescribing initiatives was correlated with pharmacists' assertiveness and confidence, yet persistent barriers such as lack of support, resources and education and fear of negative consequences, limit their translation into practice.^33^, ^34^ An exploratory qualitative study involving healthcare professionals, including 14 pharmacists, also showed that “guidance on the approach and execution of opioid deprescribing” to help ease the “cognitive load and shift the default behaviour from prescribing to deprescribing” may also support deprescribing practices.^16^ Our findings, therefore, highlight the need for strategies that address practice‐related barriers as well as structural and systemic barriers to strengthen pharmacists' role in supporting deprescribing in clinical practice.

With respect to medicinal cannabis, pharmacists demonstrated lower levels of comfort and confidence compared with other medication classes, with less than two‐thirds feeling comfortable intervening when concerned about medicinal cannabis prescriptions and only about one‐third confident in discussing deprescribing with patients presenting medicinal cannabis prescriptions. These findings align with earlier surveys, which showed that pharmacists recognized their role in facilitating safe access to medicinal cannabis, but often felt uncomfortable, and lacked the knowledge and confidence in delivering education and support strategies related to medicinal cannabis use.^35^ Specifically, a 2022 systematic review found the lack of confidence appeared to be driven by “limited understanding of medicinal cannabis, from pharmacotherapy to legislation”,^36^ highlighting pharmacists' knowledge gap as a key barrier to practice. This may also reflect the limited and evolving evidence base for medicinal cannabis and the lack of clinical guidelines for various indications that medicinal cannabis can be prescribed for.^37^, ^38^ Given the increasing prescribing of medicinal cannabis, coupled with the high prevalence of medicinal cannabis use disorder (28.4%) among people using cannabis for medical reasons, there is an urgent need for targeted training and support to strengthen pharmacists' knowledge, confidence and competence in this area, to enhance their engagement in deprescribing practices and contribute to safer medication use.^8^, ^39^, ^40^

Pharmacists working outside of capital cities and more experienced pharmacists (i.e. pharmacy managers/owners and those with ≥15 years of professional experience) had higher odds of initiating discussions on deprescribing psychotropic medicines. Given that a greater proportion of the Australian population resides in capital cities, the lower likelihood of initiating deprescribing discussions in these settings is concerning. However, higher rates of medication‐related harm in regional and remote areas may partly explain the greater likelihood of deprescribing discussions observed in these locations.^41^ Furthermore, more experienced pharmacists may be more comfortable and confident in providing information and engaging in discussions about deprescribing, as they are likely to have longer‐standing relationships and stronger rapport with their patients.^42^ Similarly, pharmacists practicing outside of capital cities may benefit from greater continuity of care, which fosters rapport and trust, whereas in capital cities, more choices of pharmacies for patients may reduce continuity of care and limit opportunities for pharmacists to engage in sustained deprescribing conversations. Targeted education and training initiatives aimed at less experienced pharmacists or those working in capital cities could, therefore, enhance pharmacists' engagement in deprescribing. Further qualitative research may identify specific types of training and support that improve pharmacists' confidence and comfort in deprescribing and explore other contextual factors that may facilitate or limit deprescribing practices in these settings.

Opioid‐specific interventions, including opioid health literacy resources such as the Opioid Safety Toolkit,^31^ and real‐time prescription drug monitoring programs, were positively correlated with pharmacists initiating discussions on opioid deprescribing. The availability of health literacy resources has been shown to increase the provision and uptake of take‐home naloxone (an opioid antagonist that can reverse the effects of an opioid overdose), and facilitate healthcare provider discussions among adults prescribed opioids for pain.^31^ Our findings reinforce that these tools can enhance pharmacists' engagement in deprescribing conversations, likely through empowering patients and providing resources for pharmacists to have patient‐centred discussions about their medication use.^43^ However, comparable toolkits for other high‐risk psychotropic medicines, such as benzodiazepines and gabapentinoids, are currently lacking. Developing and implementing similar resources for these medicine classes may enhance pharmacists' capacity to engage in broader deprescribing efforts. Furthermore, pharmacists who reported consistent use of their state's prescription drug monitoring program had 50% higher odds of initiating discussions on opioid deprescribing compared to those who used the system less frequently. This is consistent with prior literature that suggests the value of prescription drug monitoring programs not only as surveillance tools but also as clinical enablers that support informed clinical decision making and timely interventions by pharmacists.^30^, ^44^, ^45^ Strengthening the integration of prescription drug monitoring programs into routine pharmacy practice, alongside targeted educational initiatives, may further increase proactive deprescribing practices across multiple high‐risk psychotropic medicine classes.

### 5.1 Strengths and limitations

This sample included more than 12% of all Australian community pharmacies, with proportional representation across all states and territories and diversity of geographic settings from capital cities to remote areas. The survey was conducted using a robust process involving stakeholder consultation, systematic recruitment and a data collection process, informed by validated methods reported in prior literature.^26^, ^27^, ^46^

The following limitations should be acknowledged. First, due to the nature of recruitment and data collection in conducting our survey, there is a potential for self‐selection bias. For example, it is possible that pharmacists with stronger interests in harm reduction, or who were more motivated to contribute to service improvement initiatives and research activities, were overrepresented among participants. In addition, there is a potential for self‐reporting bias, particularly in relation to pharmacists' reported comfort and confidence in their practice. However, the use of an anonymous survey may have helped to mitigate this bias, and low confidence reported for some medication classes suggests that the extent of overestimation is likely to be minimal. Second, the survey did not specify the clinical context in which the medicines were prescribed. For example, participants were asked about opioid analgesics, benzodiazepines, gabapentinoids and medicinal cannabis generally, without reference to their indications (i.e. opioids for chronic pain *vs*. malignancy, or gabapentinoids for neuropathic pain *vs*. seizures). This limits our ability to interpret pharmacists' deprescribing practices relative to the specific clinical scenarios. However, given that most of these medicines are typically recommended for short‐term use (e.g. opioids for non‐cancer pain, benzodiazepines for insomnia and gabapentinoids for acute pain and anxiety disorders^47^), the overall impact on our results may be modest. Third, the use of quantitative survey data limited the exploration of more nuanced factors such as stigma and other contextual influences, which may be better explored through future qualitative research. Fourth, the survey was conducted among Australian community pharmacists, which may limit direct applicability of our findings to other countries, particularly those with different regulatory frameworks and professional scopes of practice. However, Australia shares similarities with other high‐income countries, such as the US, UK and Canada, in terms of pharmacy education and competencies,^48^ and the trends and practice gaps highlighted in this study are likely to resonate with global efforts to expand the role of community pharmacists, especially in countries where initiatives around pharmacist deprescribing are emerging or already established.^21^, ^49^ Our study findings, therefore, contributes to the evolving landscape of pharmacist deprescribing and may inform the broader international conversation about the future of pharmacy services.^50^

## 6 CONCLUSION

This study is one of the first to describe pharmacists' practices, confidence and perspectives around the provision of four key classes of high‐risk psychotropic medicines and their deprescribing in community pharmacy settings. Notably, pharmacists reported high confidence and familiarity with opioids, and appeared relatively ready to intervene in their use, whereas confidence was much lower for other medicines, such as medical cannabis. This disparity underscores an important opportunity to up‐skill pharmacists through targeted education, training and evidence‐based resources to support safe and effective deprescribing across a broad range of high‐risk psychotropic medicines. These trends and identified gaps may inform future research and efforts to advance and expand community pharmacists' roles in deprescribing internationally. Future studies could explore the effectiveness of pharmacist‐led deprescribing interventions in reducing medicines‐related harms and improving patient health outcomes in Australia.

## AUTHOR CONTRIBUTIONS

MJ, LP and SN contributed to the study conception and design. Data collection was performed by MJ, LP, RL and JD and analysis were performed by MJ and LP. The first draft of the manuscript was written by MJ and all authors commented on previous versions of the manuscript. All authors read and approved the final manuscript.

## CONFLICT OF INTEREST STATEMENT

The authors declare that there are no relevant financial or non‐financial competing interests to report.

## Data Availability

The datasets generated and analysed during the current study are available from the corresponding author upon reasonable request.
